# Targeting Dysregulated Lipid Metabolism in Cancer with Pharmacological Inhibitors

**DOI:** 10.3390/cancers16071313

**Published:** 2024-03-28

**Authors:** Amogh Gupta, Dipanwita Das, Reshma Taneja

**Affiliations:** Department of Physiology, Healthy Longevity and NUS Centre for Cancer Research Translation Research Program, Yong Loo Lin School of Medicine, National University of Singapore (NUS), 2 Medical Drive, MD9, Singapore 117593, Singapore

**Keywords:** lipid signalling, cancer stem cells, metastasis, epigenetics, therapeutics

## Abstract

**Simple Summary:**

Lipid metabolism involves signalling pathways that control the uptake, synthesis, and breakdown of lipid molecules. This highly controlled system is dysregulated in cancer, aiding new membrane synthesis, energy production, and the ability to survive oxidative stress. The epigenome of cancer cells contributes to this dysregulation. Here, we delve into the dysregulation of lipid metabolism across diverse cancer types and examine how particular facets such as fatty acid uptake, synthesis, and oxidation contribute to oncogenesis. We also discuss the role of epigenetic modulators in these processes and review metabolic drugs in cancer therapeutics.

**Abstract:**

Metabolic plasticity is recognised as a hallmark of cancer cells, enabling adaptation to microenvironmental changes throughout tumour progression. A dysregulated lipid metabolism plays a pivotal role in promoting oncogenesis. Oncogenic signalling pathways, such as PI3K/AKT/mTOR, JAK/STAT, Hippo, and NF-kB, intersect with the lipid metabolism to drive tumour progression. Furthermore, altered lipid signalling in the tumour microenvironment contributes to immune dysfunction, exacerbating oncogenesis. This review examines the role of lipid metabolism in tumour initiation, invasion, metastasis, and cancer stem cell maintenance. We highlight cybernetic networks in lipid metabolism to uncover avenues for cancer diagnostics, prognostics, and therapeutics.

## 1. Introduction

All living organisms have the innate capacity to extract energy from their surroundings for survival. Metabolism involves biochemical processes that convert molecules into energy. Metabolic processes include catabolism and anabolism. Catabolism involves the breakdown of complex biomolecules like carbohydrates, fats, and proteins into smaller molecules such as glucose, fatty acids, and amino acids, which are used to generate energy through cellular respiration. Anabolism, on the other hand, involves the synthesis of complex molecules important for cellular functions, like proteins, lipids, and nucleic acid, from simpler precursors, and it requires energy from the cell. The balance between anabolism and catabolism is maintained by a highly controlled network of enzymes, hormones, and signalling molecules. In eukaryotic cells, lipid metabolism is critical for energy production via β-oxidation in the mitochondria, membrane synthesis, and cell signalling. In this review, we provide state-of-the-art information on lipid signalling pathways and their roles in cancer progression and the maintenance of cancer stem cells. While deregulation of lipid metabolism in oncogenesis is now gaining increasing attention, there has been limited progress in a translation to therapy. We underscore prognostic, diagnostic, and therapeutic avenues that have come to light through regulatory networks and epigenetic modifiers that regulate lipid metabolism genes. This understanding will serve as a guide for novel therapeutic avenues in this rapidly evolving field.

## 2. Overview of Lipid Metabolism

Lipid metabolism includes the synthesis, breakdown, and transport of lipid molecules. The major pathways involved include fatty acid synthesis, fatty acid oxidation (FAO), triacyl glycerol (TAG) synthesis and degradation, phospholipid synthesis and remodelling, cholesterol synthesis, and lipoprotein metabolism [1]. We briefly discuss each of them in the following section.

### 2.1. Fatty Acid Synthesis

**Figure 1:**
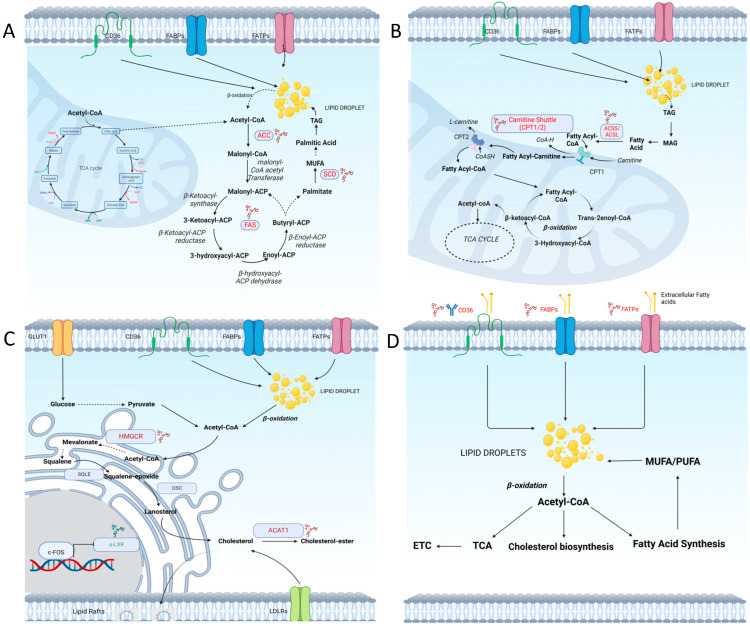
Lipid metabolism targeting for cancer therapy. (**A**). Fatty acid synthesis. (**B**). Fatty acid oxidation. (**C**). Cholesterol metabolism. (**D**). Fatty acid uptake. Molecules highlighted in red show potential targetable leads for dysregulated lipid metabolism in cancers.

Fatty acid synthesis involves the conversion of the intermediate acetyl-CoA to fatty acid (Figure 1A). In a chain of reactions, acetyl-CoA is converted to malonyl-CoA by the enzyme acetyl-CoA carboxylase (ACC). Malonyl-CoA provides the two carbon units that are condensed by fatty acid synthase (FASN) and in a stepwise manner yields palmitic acid (16:0), a 16 carbon-saturated fatty acid as the end product. Fatty acids are initially synthesised saturated and then undergo de-saturation by the activity of the enzymes stearoyl-CoA desaturase (SCD) and delta-5 and delta-6 desaturases, which are rate-limiting desaturases in this step. Cells synthesise fatty acids of varied lengths and degrees of unsaturation with the help of this complex system of enzymes. Fatty acid synthesis is an energy-intensive process that requires ATP, NADPH, and other cofactors. It is regulated by a variety of factors, including the availability of substrates, the activity of FAS and other enzymes, and hormonal signals such as insulin [2,3]. 

The ω-6 series derived from cis-linoleic acid (LA, 18:2) and the ω-3 series derived from α-linolenic acid (ALA, 18:3) are essential fatty acids (EFAs) that must be obtained through the diet and cannot be synthesised. These poly-unsaturated fatty acids (PUFAs) are either acted on by enzymes in the body to produce a range of signalling/inflammatory molecules like arachidonic acids or eicosanoids (prostaglandins, leukotrienes), or they can be directly involved in various physiological processes. EFAs play an important role in controlling membrane fluidity, pathways like TNF-α, inflammation, and cytokine signalling processes and are implicated in various diseases [4].

### 2.2. Fatty Acid Oxidation (FAO)

The breakdown of fatty acid takes place by a series of oxidation steps that remove 2 carbon molecules from the fatty acid chain. Initially, the fatty acid moiety is activated by conjugation with coenzyme A (CoA) to form fatty acyl-CoA, catalysed by the enzyme fatty acyl-CoA synthase. Fatty acyl-CoA is then transported to the mitochondria for β-oxidation by transporters carnitine palmitoyl transferase I and II (CPT1, CPT2). In the mitochondria, fatty acyl-CoA is subjected to a series of cyclic enzymatic reactions that remove two carbons at a time from the fatty acid chain and yield acetyl-CoA. This cycle continues until the entire fatty acid chain is broken down into acetyl-CoA molecules. The acetyl-CoA produced is shunted back to the TCA cycle to generate ATP (Figure 1B). β-oxidation is regulated by several factors, including the availability of fatty acids, hormonal regulation, and the energy status of the cell. As each fatty acid can produce acetyl-CoA, the process of FAO produces ATP and provides the cell with energy under stress [5].

### 2.3. Triacyl Glycerol Synthesis and Degradation

Fatty acids are esterified with glycerol to form triacylglycerol (TAG). TAG synthesis occurs in the endoplasmic reticulum and is stored in lipid droplets within the adipose tissue. The process begins with the phosphorylation of glycerol to glycerol-3-phosphate (G3P) catalysed by the enzyme glycerol kinase. G3P is esterified with fatty acid to form lysophosphatidic acid (LPA) by glycerol-3-phosphate acyltransferase (GPAT) activity. Another step of esterification with the fatty acid chain produces phosphatidic acid (PA). PA is dephosphorylated by the enzyme phosphatidic acid phosphatase (PAP), also known as lipin, to produce diacylglycerol (DAG). Finally, DAG is esterified with a third fatty acid by the enzyme diacylglycerol acyltransferase (DGAT) to produce TAG [6].

During periods of energy demand, TAGs are hydrolysed by lipases to release free fatty acids and glycerol, which can be used for energy production. This process is initiated by the activation of lipases, a group of enzymes that catalyse the hydrolysis of TAGs. Hormone-sensitive lipase (HSL) and adipose triglyceride lipase (ATGL) are involved in TAG degradation. The activity of these enzymes is regulated by hormonal signals, such as glucagon, adrenaline, and insulin. The activity of triglyceride lipase generates three free fatty acids and a glycerol moiety. The released free fatty acids can be transported to other tissues, such as the liver or skeletal muscles, where they undergo β-oxidation to produce energy. Glycerol is taken up by the liver and converted back into glycerol-3-phosphate or used as a substrate for gluconeogenesis [7].

### 2.4. Phospholipid Synthesis and Remodelling

Phospholipids are synthesised in the endoplasmic reticulum and golgi apparatus from G3P, fatty acids, and a polar head group (e.g., choline, serine, or inositol). It follows the same steps discussed in the above section, where G3P is acted upon in a series of enzymatic steps to form DG or cytidine diphosphate diacylglycerol (CDP-DG) by the action of the enzyme CDP-DG synthetase. DAG and CDP-DG is then combined with a polar head group (activated with cytidine diphosphate CDP) via a reaction catalysed by specific phospholipid-synthesizing enzymes to make phospholipids. 

Phospholipid remodelling, also known as the Lands cycle, involves the exchange and modification of fatty acids within existing phospholipids to regulate membrane fluidity and composition. Phospholipases (PLAs) carry out the de-acylation of phospholipids, generating a lysophospholipid (a phospholipid with only one fatty acid) and a free fatty acid. The remodelling process can be selective, favouring the incorporation of specific fatty acids, such as monounsaturated or polyunsaturated fatty acids (MUFAs) (PUFAs), which influence membrane fluidity, signalling, and other cellular functions. Saturated fatty acids are favoured in the sn-1 position for stability, while unsaturated fatty acids are preferred in the sn-2 position for flexibility on the glycerol backbone. This arrangement maintains the membrane integrity and fluidity essential for cellular functions. Enzymes like phospholipases and acyltransferases regulate fatty acid composition, ensuring an optimal balance between stability and flexibility. The balance between synthesis and remodelling is critical for maintaining membrane function and overall cellular homeostasis [8,9].

### 2.5. Cholesterol Synthesis

Cholesterol is an important lipid component of cell membranes, modulating their fluidity and permeability. Cholesterol is also a major component of lipid rafts, which are microdomains in the lipid bilayer with restricted fluidity that are rich in cholesterol, sphingolipids, and certain proteins. These microdomains play crucial roles in various cellular processes such as signal transduction, membrane trafficking, and cell adhesion [10]. Cholesterol also serves as a precursor for the synthesis of steroid hormones, bile acids, and vitamin D.

Cholesterol synthesis occurs in the endoplasmic reticulum and involves the conversion of acetyl-CoA into cholesterol through a series of enzymatic reactions, with HMG-CoA reductase being a key regulatory enzyme (Figure 1C) [11].

Cholesterol can also be acquired from dietary sources and transported through the bloodstream in lipoprotein particles. Cholesterol from the diet or synthesised in the liver is transported through the bloodstream in lipoprotein particles, such as low-density lipoprotein (LDL) and high-density lipoprotein (HDL). Cells take up cholesterol from LDL and HDL particles via LDL/HDL receptor-mediated endocytosis. Cholesterol can be transported back to the liver or other tissues by HDL particles, a process known as reverse cholesterol transport. Balancing cholesterol synthesis, uptake, and excretion is critical for maintaining cellular and systemic cholesterol homeostasis [12].

### 2.6. Lipoprotein Metabolism

Lipoproteins are complex particles that transport lipids through the bloodstream. They consist of a hydrophobic lipid core and hydrophilic surface composed of phospholipids, cholesterol, and apolipoproteins. Major classes of lipoproteins include chylomicrons, very low-density lipoproteins (VLDL), low-density lipoproteins (LDL), and high-density lipoproteins (HDL). Lipoprotein metabolism involves the synthesis, remodelling, and clearance of these particles and is essential for lipid homeostasis and transport [5].

## 3. Lipid Signalling

Lipid signalling regulates a wide range of physiological functions, including metabolism, growth, differentiation, migration, and apoptosis. These pathways involve the activation and regulation of lipid signalling molecules, such as phospholipids, sphingolipids, and eicosanoids, which act as signalling messengers to communicate between and within cells [13].

### 3.1. PI3/AKT Pathway

One of the most well-known lipid signalling pathways is the phosphoinositide 3-kinase (PI3K)/AKT pathway. This pathway is activated by a variety of extracellular signals, including growth factors, cytokines, and hormones. Upon activation, PI3K phosphorylates phosphatidylinositol-4,5-bisphosphate (PIP2) to produce phosphatidylinositol-3,4,5-trisphosphate (PIP3). PIP3 then activates the protein kinase AKT, which regulates numerous downstream targets involved in cell growth, survival, and metabolism [14,15].

### 3.2. Arachidonic Acid Pathway

The arachidonic acid (AA) pathway is involved in the production of eicosanoid signalling molecules, such as prostaglandins, leukotrienes, and thromboxanes. Arachidonic acid is released from cellular membranes by the action of phospholipaseA_2_ (PLA_2_) enzymes and is then converted into various eicosanoids by the cyclooxygenase (COX), lipoxygenase (LOX), and cytochrome P450 enzymes. Eicosanoids play important roles in regulating inflammation, blood clotting, and immune responses and have been implicated in cancer modulating, effecting tumour growth and effecting the microenvironment [16,17,18].

### 3.3. Sphingolipid Pathway

Sphingolipids, including sphingosine and ceramide, act as signalling messengers to regulate a wide range of cellular functions, such as cell growth, differentiation, and apoptosis. Sphingolipid signalling is regulated by the activity of sphingosine kinases, which phosphorylate sphingosine to produce sphingosine-1-phosphate (S1P), and by the activity of sphingomyelinases, which generate ceramide from sphingomyelin [19].

### 3.4. AMPK Pathway

Lipid signalling pathways are also involved in the regulation of energy metabolism. The AMP-activated protein kinase (AMPK) pathway is stimulated by increased intracellular AMP levels in response to energy depletion. AMPK regulates fatty acid synthesis and oxidation, glucose uptake, and mitochondrial biogenesis [20,21].

In the following sections, we discuss the dysregulation of lipid metabolism and the therapeutic opportunities of these pathways in oncology.

## 4. Altered Lipid Metabolism in Cancer and Therapeutic Avenues

Dysregulation of lipid metabolism is a hallmark of cancer, contributing to cancer cell proliferation, invasion, and resistance to therapy. Lipid metabolic alterations in cancer cells include the following:

### 4.1. Increased Fatty Acid Uptake

An increased uptake of fatty acids is a common feature of many types of cancer, including breast, prostate, and pancreatic cancer [22,23]. The uptake of fatty acids is regulated by the expression of fatty acid transporters, such as CD36, FABP4, and FATP1, and the activity of lipases that release fatty acids from circulating lipids. Fatty acid uptake increases the reservoir of lipid droplets which feed into different metabolic pathways, such as energy production and cholesterol synthesis, or are repurposed into other fatty acids by modifications (Figure 1D). Disrupting the uptake can affect these networks in cancer cells.

**CD36**: CD36 is overexpressed in several types of cancer [24]. CD36+ oesophageal cancer cells show enhanced FAO and the ability to survive in stressed conditions. These cells are more migratory and contribute to metastasis [25]. CD36-mediated increased fatty acid uptake disrupts the lipid metabolic balance, leading to the accumulation of signalling lipids such as acyl carnitines (ACS), monoacylglycerols (MAGs), and various acyl and ether lysophospholipids. These signalling lipids play critical roles in pathways associated with fatty acid oxidation and lysophospholipid-mediated signalling cascades, including the Rho, PI3K/AKT, and MAPK pathways. In prostate cancer, the uptake of fatty acid is increased through a high CD36 expression, and treatment with the CD36 mAB antibody significantly reduces tumour volume [22]. In oral carcinoma, CD36+ cells initiate metastasis, which is enhanced with a high fat/palmitic acid diet in orthotopic mouse models [26]. CD36 plays an important role in the tumour microenvironment as well, where it enhances the function of tumour-associated macrophages and aids in tumour proliferation [27].

Antibodies that target fatty acid uptake bind to fatty acid transporters and inhibit their function (Figure 1D) (Table 1). They also prime cancer cells for an immune response. The murine antibody JC63.1 is a potent agent in altering tumour initiation and progression by effectively modifying lipid uptake and reducing stemness and migratory capability in bladder cancer cells and gastric cancer [28]. Targeting CD36+ on tumour-infiltrating lymphocytes leads to an enhanced CD8+ T cell effector function in melanoma models [29]. Humanised CD36 antibodies, exemplified by clones 1G04 and Ona-0-v1, mirror the efficacy of JC63.1 and diminish tumour initiation and metastasis, particularly in liver and lung cancer models, by modulating lipid uptake [30]. Small-molecule inhibitors targeting CD36, like Sulfosuccinimidyl oleate (SSO), significantly curtail fatty acid uptake, leading to the inhibition of ovarian cancer cell growth and the reduced migration of hepatocellular carcinoma (HCC) cells [31,32]. Another inhibitor, 2-methylthio-1,4-naphthoquinone (MTN), plays a role in suppressing lipid uptake and reducing the population of cancer stem cells in glioblastoma multiforme [33].

**FABP4**: FABP4 promotes cancer cell proliferation, motility, angiogenesis, and resistance to chemotherapy, and it also inhibits antitumour immunity. High lipoprotein-A and visceral fat are key risk factors indicating the expression of FABP4 in CRC patients, where FABP4 promotes tumour angiogenesis [34]. In non-small-cell lung cancer (NSCLC), both FABP3 and FABP4 are upregulated and positively correlated with the advanced tumour node metastasis stage (TNM) across matched cancerous and non-cancerous tissue from 30 patients [35]. In gastric cancer, FABP4 promotes tumour growth by discouraging the survival of tissue-resident memory T cells, as they cannot compete with cancer cells for fatty acid uptake [36]. A CRISPR-mediated knockdown of FABP4 resulted in a significant decrease in the metastatic tumour burden and FABP4 inhibition using BMS309403-sensitised ovarian cancer cells to carboplatin, indicating that lipid uptake mediated by FABP4 contributes to chemoresistance in this system [37,38]. In prostate cancer, exogenous FABP4 promotes tumour growth and metastasis by activating the PI3K/AKT and MAPK/ERK pathways [39]. In non-small-cell lung cancer (NSCLC), both FABP3 and FABP4 are upregulated and promote the tumorigenicity of NSCLC cells [35]. Similarly, in cervical cancer, an upregulated FABP4 expression promotes invasion and metastasis by inducing an epithelial-to-mesenchymal transition (EMT), where epithelial cells acquire mesenchymal properties, leading to increased motility, invasion, and metastasis [40].

BMS309403, a biphenyl azole compound [41], effectively reversed the enhanced invasiveness induced by exogenous FABP4 in prostate cancer [39]. Additionally, BMS309403 treatment in colon cancer led to a reduced lipid accumulation and decreased invasive potential in vitro [42]. In hepatocellular carcinoma (HCC), BMS309403 curtailed tumour growth by downregulating key cell cycle genes (such as cyclin D1), VEGFA, and VEGFR expression, while inducing active caspase 3 [43]. In ovarian cancer, BMS309403 reversed adipocyte-induced aggressiveness, resulting in a reduced tumour burden [38]. Another promising FABP4 inhibitor, BD62694, exhibited efficacy by reducing the tumour burden and lung metastasis in prostate cancer [39] (Table 1).

**FATP1**: FATP1 is a member of the FATP family of membrane proteins that facilitate the cellular uptake of long-chain fatty acids (Figure 1A). In prostate cancer, FATP1 expression is upregulated, and its inhibition suppresses growth and sensitises cells to FAO inhibition [44]. In breast cancer, a high FATP1 expression is associated with a poor prognosis, and its inhibition impairs cancer cell viability. ER-β regulates FATP1 expression [45]. In lung cancer, FATP1 expression is increased upon benzo[a]pyrene (BaP) exposure, where it contributes to the epithelial–mesenchymal transition (EMT) in these cells by aryl hydrocarbon (AHR) signalling, increasing various EMT proteins’ expression like SNAI2, TWIST1 and 2, and E-cadherin. PPAR-γ, which is upregulated in many cancers, was decreased in BaP-exposed cells, with increased activity of FATP1 [46].

In preclinical studies, the inhibition of FATPs with lipofermata is effective in abrogating melanoma cell invasiveness and tumorigenicity [47]. A specific inhibitor of FATP1, arylpiperazine 5k (DS22420314), has demonstrated promise [48]. In breast cancer cells, where FATP1 expression is elevated, inhibition with arylpiperazine 5k leads to a decrease in proliferation, migration, and invasion, accompanied by increased apoptosis. Importantly, FATP1 expression is regulated by estrogen receptor β (ER-β), suggesting that targeting FATP1 with arylpiperazine 5k could be a potential treatment for ER+ breast cancer [45]. Co-culturing breast cancer cells with cancer-associated fibroblasts (CAFs) results in an increased expression of genes involved in lipid metabolism, including FATP1. This indicates that targeting the metabolic crosstalk between CAFs and breast cancer cells, including the inhibition of FATP1, offers a potential therapeutic strategy [49]. In aggressive B cell lymphoma, characterised by a metabolic switch to fatty acid metabolism and high FATP1 activity, inhibiting FATP1 with arylpiperazine 5K leads to a decrease in the tumour burden and invasiveness [50] (Table 1).

### 4.2. Increased Fatty Acid Synthesis

Cancer cells exhibit an increased demand for lipids, which can be met by either uptake from the extracellular milieu or by de novo synthesis within the cell. While an increased uptake of exogenous lipids is often observed in many cancers, de novo lipid synthesis also plays a critical role in promoting tumour growth and survival. Fatty acid synthesis enzymes, including ACC, FASN, and SCD, are upregulated in various cancers and are associated with cancer progression. They catalyse crucial steps in the de novo lipogenesis processes and can repurpose metabolites like acetyl-CoA to support the fast-growing neoplasm with a range of important FAs (Figure 1A).

**ACC**: ACC is upregulated in various cancer types, including AML, lung, liver, and breast cancer, and a high expression is associated with a poor prognosis [51,52]. ACC is involved in oncogenic pathways like the PI3K/AKT/mTOR pathway and acts as its effector in HER2+ breast cancer, along with FASN [53]. ACC promotes breast cancer cell proliferation and survival and interacts with BRCA1, indicating links between fatty acid synthesis and the genetic factors involved in breast cancer susceptibility [54]. It also regulates EMT [52]. In hepatocellular carcinoma (HCC), ACC is positively correlated with tumour size, differentiation, and vascular invasion [55]. ACC inhibition reduced the proliferation and migration of HCC cells. ACC plays a role in the glucose metabolism in cancer cells, where it drives glucose-derived lipogenesis [56]. Additionally, ACC inhibition sensitises HNSCC cells to cetuximab treatment, which is targeted to block the upregulated glycolysis in these cells. HNCC cells resistant to cetuximab treatment rely on ACC activity to rewire the metabolism [57].

Soraphen A is a potent inducer of cell death in prostate cancer cells, demonstrating efficacy even at nanomolar concentrations [58]. In acute myeloid leukaemia (AML), the drug combination Bezafibrate and Medroxyprogesterone Acetate (BaP) exhibits antitumour activity by disrupting monounsaturated fatty acid synthesis [59,60]. ND-646, an allosteric inhibitor of ACC, exhibits potential as an adjuvant therapy in lung cancer cells, adding to the arsenal of ACC-targeted treatments [61]. A liver-specific ACC inhibitor, ND-654, mimics the effect of ACC1 phosphorylation, resulting in decreased hepatocellular carcinoma (HCC) development and increased survival in tumour-bearing mice, either alone or in combination with the multi-kinase inhibitor sorafenib [55]. Additionally, the structural inhibitor of ACC, 5-Tetracepoxy-2-Furan Acid (TOFA), is effective in inhibiting ACC activity. In head and neck squamous cell carcinoma (HNCC), TOFA treatment shows a significant decrease in cell proliferation when combined with cetuximab in cetuximab-resistant cells [57] (Table 1).

**FASN**: FASN is overexpressed in various types of cancers [62], where it contributes to the increased production of fatty acids (Figure 1B). An elevated FASN expression is associated with a poor patient prognosis in colorectal cancer [40,41], and in melanoma, prostate cancer, and lymphoma, it promotes tumour growth and survival [43,44,45]. In pancreatic cancer, FASN is linked to therapy resistance against gemcitabine and radiation.

The inhibition of FASN expression in NSCLC reduced proliferation and motility, with downregulated AKT/ERK signalling [63]. In radiotherapy-resistant prostate cancer, a combination of FASN inhibition and radiotherapy decreased NF-kB activity and induced apoptosis [64], whereas in breast tumours, apoptosis was concomitant with pro-death BH3 family proteins BIM, PUMA, and NOXA [65].

A range of FASN inhibitors have now been implicated for cancer therapy (Table 1). Cerulenin is a FASN inhibitor, known for inducing apoptosis and inhibiting tumour growth in preclinical studies, It demonstrated efficacy in colon cancer, leading to a marked decrease in liver metastasis with reduced pAkt levels in a mouse model of colon cancer [66]. In cisplatin-resistant NSCLC cells, cerulenin reduced the metastatic potential by targeting the EMT regulated by FASN, without any impact on tumour growth, which was confirmed in an orthotropic model [67]. Another FASN inhibitor, C75, decreased lymph node metastasis in cervical cancer and reduced the tumour burden in lung adenocarcinoma, albeit with a significant drop in mouse body weight [68,69]. Notably, C75 uniquely sensitised prostate cancer cell lines to ionizing radiation, and its effects were synergistically increased when combined with an anti-CD36 antibody [44]. Orlistat, another FASN inhibitor, demonstrated cytotoxic effects in various cancer cell lines and xenograft models [70]. However, these compounds faced limitations due to poor pharmacokinetics and toxicity issues. The development of the second-generation FASN inhibitors, TVB-2640 and GSK2194069, addressed these challenges with improved profiles [71]. These inhibitors have yielded promising results in preclinical studies, effectively inhibiting tumour growth and metastasis in several cancer types. TVB-2640 advanced to clinical trials for advanced solid tumours, including breast, colon, and ovarian cancer [72]. Notably, in recent Phase 2 clinical studies, TVB-2640 paired with bevacizumab in high-grade astrocytoma demonstrated minimal adverse effects and excellent patient tolerability [73].

**SCD**: SCD is upregulated in many cancers, including androgen receptor (AR)+ LNCaP prostate cancer cells [74], ErbB2-driven breast cancer cells [75], pancreatic cancer cells [76], glioblastoma cells [77], non-small-cell lung cancer patient samples (NSCLC) [78], and gastric cancer cells [79]. It is associated with poor patient survival and prognosis and supports tumour growth, survival, migration and invasion capabilities. One proposed mechanism is that SCD promotes the synthesis of monounsaturated fatty acids (MUFAs), such as oleic acid, from saturated fatty acids (SFAs) in cancer cells (Figure 1A). MUFAs stimulate the growth of cancer cells and inhibit apoptosis. Additionally, MUFAs activate signalling pathways that promote cell proliferation and survival, such as the Wnt signalling and NOTCH pathways [80]. The increase in fatty acid synthesis in cancer cells leads to alterations in the lipid composition of cell membranes, including an increase in saturated and monounsaturated fatty acids. These alterations affect the fluidity and stability of cell membranes and contribute to aggressiveness, enhanced migration, invasion, and metastasis.

An inhibition of stearoyl-CoA desaturase (SCD) through various approaches, including siRNAs and small-molecule inhibitors, has demonstrated efficacy in reducing tumour growth and metastasis across a spectrum of cancer types such as endometrial, bladder, thyroid, colon, glioblastoma, prostate, and lung cancers [81,82]. The development of small-molecule inhibitors, such as A-939572 and CAY10566, has yielded promising results in preclinical studies, effectively inhibiting tumour growth and metastasis in lung and ovarian cancer models, respectively [83,84]. Additionally, the SCD inhibitor CVT-11127 induces cell cycle arrest in H460 lung cancer cells without impairing the proliferation of normal human fibroblasts [85,86] (Table 1).

### 4.3. Altered Fatty Acid Oxidation

Cancer cells alter FAO to support their survival and growth. FAO promotes the production of reactive oxygen species (ROS), which contribute to cell proliferation and survival [87,88]. FAO also contributes to resistance to therapy. For example, in breast cancer stem cells (CSCs), FAO plays a major role in sustenance and resistance to therapy driven by the JAK/STAT3 pathway. It provides the cancer cell with much-needed energy at times of stress by utilising the energy stored in the acyl chains in FAs (Figure 1B).

Blocking FAO or depleting leptins inhibits self-renewal and leads to the sensitisation of cells to chemotherapy (Table 1). In triple-negative breast cancer (TNBC), FAO results in STAT3 acetylation, which leads to the upregulation of long-chain acyl-CoA synthetase 4 (ACSL) [89]. This helps bolster mitochondrial membrane integrity to overcome chemotherapy-induced apoptosis. 

**Carnitine palmitoyltransferase (CPT)** facilitates the transport of long-chain fatty acids across the mitochondrial membrane, where they undergo β-oxidation to generate acetyl-CoA and produce ATP (Figure 1C). In breast cancer, the stabilisation of CPT1A mRNA by IGF2BP1 leads to an increase in cell migration and invasion [90]. The inhibition of CPT1A expression decreases proliferation and tumorigenicity in melanoma cells [91]. CPT1A upregulation in ovarian cancer promotes anoikis, and the inhibition of CPT1A leads to decreased cancer cell proliferation and migration [92]. In prostate cancer, CPT1A promotes cell survival by modulating ROS production in the mitochondria, by upregulating superoxide dismutase 2 (SOD2) [93].

Etomoxir, a well-established CPT1A inhibitor, demonstrates notable antitumour effects across various cancer models. It exhibits efficacy in decreasing the growth and proliferation of endocrine-resistant breast cancer cells by inhibiting FAO [94]. In prostate cancer, etomoxir suppressed cell growth by reducing both FAO and androgen receptor expression levels [93]. Etomoxir proved effective in colorectal cancer, where it hinders anoikis resistance and metastasis by curbing FAO [95]. Another CPT1A inhibitor, Perhexiline, showed promising antitumour effects in hepatocellular carcinoma (HCC) cells [96]. Chronic lymphocytic leukaemia (CLL) cells, characterised by high CPT1 expression, exhibit an enhanced sensitivity to perhexiline compared to lymphocytes [97]. Additionally, the novel CPT1A inhibitor ST1326 demonstrated potent anticancer activity in preclinical models, inhibiting FAO, inducing apoptosis, and causing mitochondrial damage in patient-derived leukaemia cells (AML, ALL, CLL) [98] (Table 1).

**Acetyl-CoA synthetase (ACSS)**, also known as acetate-CoA ligase (ACL), converts acetate into acetyl-CoA. ACL has been implicated in cancer due to its role in FAO. Acetyl-CoA synthetase 2 (ASS2) activity is increased under low oxygen and lipid levels in breast cancer cells in the form of a stress response [99]. ASSS2-mediated acetate utilisation supplies cells with a pool of carbon within the fatty acid and phospholipid pool. ACSS expression is dramatically increased in bladder cancer tissues and is associated with cis-platin resistance [100]. Additionally, ACSS1 and 2 are effectors for 4-hydroxytamoxifen (4-OHT), which can induce metabolic changes and promote cell survival in ER+ breast cancer. ACSS silencing reduces the proliferation of breast cancer, where the depletion of ACSS1/2 leads to a significant decrease in cell viability [101]. In obesity-associated multiple myeloma, ACSS2 stabilises interferon regulatory factor 4 (IRF4) and aids in gene transcription through acetylation [102]. In renal cell carcinoma (RCC) ACSS2 is upregulated, interestingly, its inhibition had no effect on cell proliferation or apoptosis but led to a marked decrease in cell migration and invasion [103].

The inhibition of acetyl-CoA synthetase (ACSS2) and long-chain acyl-CoA synthetase 4 (ACSL4) has resulted in a marked decrease in tumour growth and therapeutic sensitivity in various cancers (Table 1). The novel ACSS2 inhibitor, VY-3-135, stands out for its ability to impair triple-negative breast cancer (TNBC) growth [104]. In bladder cancer, the inhibition of ACSS using 1-(2,3-di(thiophen-2-yl) quinoxalin-6-yl)-3-(2-methoxyethyl) urea sensitises cisplatin-resistant cells, expanding the therapeutic options for this challenging condition [100]. Additionally, ACSL4 knockout or knockdown in human breast cancer cells not only inhibits tumour growth and metastasis in vivo but also enhances sensitivity to doxorubicin [105,106]. Ongoing preclinical studies are exploring additional small-molecule inhibitors of ACSL4, including rosiglitazone, a PPAR-γ agonist, which has been reported to inhibit ACSL4 in PPAR-γ, to unravel their therapeutic potential in cancer treatment [107,108].

### 4.4. Dysregulated Cholesterol Metabolism

High cholesterol levels are linked to cancer progression and a poor prognosis in several cancers. An altered cholesterol metabolism contributes to cancer through the upregulation of the mevalonate pathway, which is involved in cholesterol biosynthesis (Figure 1C) [109]. This pathway also produces intermediate metabolites that are important for other cellular processes, such as protein prenylation and the synthesis of ubiquinone, a key component of the electron transport chain. 

**HMGCOA**: 3-hydroxy-3-methylglutaryl-CoA synthase 1 (HMGCS1) and 3-hydroxy-3-methylglutaryl-CoA reductase (HMGCR) are upregulated in stromal cells around prostate cancer. The inhibition of the mevalonate pathway through the knockdown of either HMGCS1 or HMGCR resulted in reduced cell viability in prostate cancer [110]. The mevalonate pathway is also upregulated in breast, lung, and hepatocellular cancer cells, and inhibition leads to decreased cell proliferation [111]. In many cancer cells, HMG-CoA reductase, responsible for the synthesis of cholesterol and other isoprenoids, is upregulated. This leads to increased cholesterol synthesis, which provides building blocks for membrane biogenesis, and isoprenoids, which have critical roles in cell signalling and protein synthesis. 

Previous studies have pointed to the fact that RAS/RAC GTPase signalling regulated myc phosphorylation, although the exact mechanism still needs to be elucidated [112]. The inhibition of HMG-CoA reductase with statins blocks farnesyl pyrophosphate (FPP) and geranylgeranyl pyrophosphate (GGPP) production. FPP and GGPP play a role in prenylating and activating the Ras and Rac family of small GTPases. This in turn effects myc phosphorylation and activation in HCC, inhibiting tumour initiation [113]. HMG-CoA reductase inhibition using fluvastatin and lovastatin induces cell death and suppresses the tumour invasion and migration of liver cancer cells [114]. Other studies have demonstrated a link between a dysregulated cholesterol metabolism and HMG-CoA reductase activity in prostate [115], lung [116], and ovarian cancer [117]. Statins, known for their cholesterol-lowering properties, effectively inhibit the proliferation and migration of cancer cells. In colorectal cancer, statin use is associated with a significant decrease in the risk of colorectal cancer [118,119]. Moreover, the use of statins has been linked to a decrease in mortality among cancer patients, emphasizing their potential impact on patient outcomes [120]. In a Danish prostate cancer patient cohort, statin usage was correlated with a decrease in motility, although the causative relationship is yet to be firmly established [121]. Notably, lipophilic statins have been found to enhance antigen presentation and immune activation in melanoma models, suggesting an additional immunomodulatory aspect to their anticancer effects [122] (Table 1).

Lipid rafts composed of cholesterol and sphingolipids sequester selected proteins, after the protein has gone through some modifications e.g., palmitoylation. Lipid rafts, driven by high cholesterol biosynthesis, can sequester proteins like CD44 that regulate cell migration and signalling [123]. Lipid rafts are frequently higher in prostate, breast [124] and melanoma tumours [125]. Interestingly, lipid rafts are higher in CSCs, and CSC markers, including CD44, CD24, CXCR4, and CD133, are frequently sequestered in lipid rafts [126,127]. The inhibition of cholesterol synthesis using statins thus also targets lipid rafts and CSCs. In pancreatic cancer, disrupting lipid rafts with lovastatin decreases the metastatic potential and chemosensitivity of CSCs in orthotropic models [128]. Cholesterol analogues like ginsenosides disrupt lipid rafts by incorporating and increasing the fluidity of the rafts. Rp1, a ginsenoside derivative, has been found to reverse multidrug resistance in ovarian cancer cells [129]. Targeting the cholesterol metabolism to disrupt lipid rafts might hold potential in cancers with CSCs and drug resistance.

Cancer cells increase cholesterol uptake by upregulating LDL receptors (LDLR) on the cell surface, which contributes to growth and proliferation. The expression of LDLR was significantly higher in breast cancer compared to adjacent normal tissue [130], and in lung cancer, it is associated with a poor prognosis [131]. Targeting LDLR leads to a cholesterol imbalance and an improvement in chemotherapy in pancreatic ductal adenocarcinoma (PDAC) [132]. In addition to upregulating LDLR expression, cancer cells also increase the uptake of cholesterol by upregulating scavenger receptor class B type I (SR-BI) that mediates the uptake of cholesterol and HDLs. Targeting HDL uptake by targeting SR-BI leads to a decrease in proliferation and migration in breast cancer cells and the downregulation of the PI3K/AKT pathway [133]. SR-BI expression is elevated in prostate cancer, and its loss reduces HDL uptake and proliferation [134]. Similarly, the depletion of SR-BI leads to a decrease in prostate-specific antigen secretion (PSA) in castration-resistant prostate cancer cells, along with a significant decrease in cell viability [135]. 

**ACAT1**: Acyl-CoA cholesterol acyltransferase 1 (ACAT1) plays a role in the esterification of cholesterol, converting free cholesterol into cholesterol esters by transferring fatty acids from acyl-CoA molecules onto the cholesterol molecule. In prostate cancer, the inhibition of ACAT1 with Avasimibe has demonstrated significant therapeutic effects [136]. Avasimibe led to a notable decrease in metastasis in both orthotopic and intracardiac injection mouse models of prostate cancer, suggesting its potential as a targeted intervention [137]. The impact of ACAT1 inhibition extends to melanoma, where Avasimibe enhances the cytotoxic response from T cells in mouse models, concurrently resulting in a decrease in metastatic dissemination and tumour growth [138]. Another ACAT1 inhibitor, Avasimin, exhibits promising anticancer properties. In prostate, pancreatic, lung, and colon cancer cell lines, Avasimin increased apoptosis. Notably, in colon and prostate cancer, this treatment translated into a decrease in the tumour burden and increased survival, emphasising the broad applicability of ACAT1 inhibition across different cancer types [139].

**LXR**: Liver X Receptor is a nuclear hormone receptor that plays a crucial role in cholesterol metabolism. There are two isoforms of LXR, LXRα (NR1H3) and LXRβ (NR1H2), which are primarily expressed in the liver, intestine, adipose tissue, and macrophages. LXR signalling with LXR agonist GW3965 led to a decrease in circulating myeloid-derived suppressor cells (MDSCs)—an immunosuppressive innate cell population [140] in different cancer types. In clear cell renal cell carcinoma, rescuing LXR signalling with agonist LXR623 led to a decrease in proliferation and tumour formation. Mechanistically, activating LXR leads to a decrease in LDR receptors and an increase in the ABC reporter (ABCA1), which leads to a decrease in intracellular cholesterol [141]. Possible leads for targeting the dysregulated lipid metabolism in cancers have been summarised in Figure 1 and Table 1.

## 5. Epigenetic Modifications Linked to Lipid Metabolism

There is a close mechanistic link between epigenetics and metabolic alterations in cancer (Table 2). Cancer cells often hijack these links. The epigenetic profile of cancer cells can be altered due to the reprogramming of the cell metabolism, which changes the availability of cofactors [142].

### 5.1. PI3K-AKT-mTORC1 Pathway, AKT–ACLY Signalling, and Histone Acetylation

The PI3K-AKT-mTORC1 pathway is associated with the production of acetyl-CoA, a chief substrate for histone acetylation [143,144]. AKT activation promotes glucose uptake and glycolysis, increasing the production of pyruvate, which is converted to acetyl-CoA through the pyruvate dehydrogenase complex (PDC). AKT also directly activates ACC, increasing malonyl-CoA production, which serves as a precursor for lipid synthesis. While this process consumes acetyl-CoA, it does not directly contribute to the pool of acetyl-CoA available for histone acetylation. The lysine acetylation of histones is sensitive to acetyl-CoA availability and is balanced by histone lysine acetyl transferases (HATs or KATs) and histone deacetylases (HDACs). AKT signalling controls histone acetylation through the modulation of ATP citrate lyase (ACLY) and the supply of acetyl-CoA [145,146,147]. In glioma and prostate and pancreatic ductal adenocarcinoma (PDAC), the oncogenic driver K-Ras upregulates AKT-ACLY signalling and histone acetylation, leading to active proliferation and enhanced tumour growth [145,148,149]. ACLY enhances the p300-dependent histone acetylation in melanoma cells, thereby promoting PPARγ coactivator (PGC) 1α-mediated mitochondrial biogenesis for cell growth [150]. ACLY ubiquitination and subsequent degradation, promoted by sirtuin-2 (SIRT2) deacetylase under high-glucose conditions, is inhibited by lysine acetyltransferase 2B (KAT2B), which mediates acetylation at K540, K546, and K554. In human lung cancers, acetylation is upregulated, which promotes tumour growth and lipogenesis [151]. Acetyl-CoA, produced by lipolysis, also facilitates the synthesis of ketone bodies like β-Hydroxybutyrate (β-HB) in the liver. Upregulated levels of β-HB, which are specific inhibitors of class I HDACs, leads to a new histone modification, lysine β-hydroxy butyrylation (Kbhb) [152,153]. Human colorectal carcinoma cells, HCT116, show β-HB-mediated p53 Kbhb at the K120, K319, and K370 sites, which leads to lower levels of p53 acetylation and consequently decreased activity, causing weakened tumour-suppressive functions [154].

### 5.2. ACCS2 and Lipid Metabolism Regulation

ACC is the master regulator of fatty acid metabolism which converts acetyl-CoA to malonyl-CoA and modulates global histone acetylation, providing a direct link between anabolic metabolism and epigenetic regulation [155]. Mammary adipocyte-derived leptin and TGFβ signalling inhibits ACC1 via TGFβ-activated kinase (TAK)1-AMPK, which leads to increased cellular acetyl-CoA and the activation of actylated-Smad2, resulting in tumour metastasis in breast cancer. Thus, the fine-tuning of ACC1 activity is essential for FA synthesis and homeostatic balance [52]. Free acetate, which is converted to acetyl-CoA, functions as an epigenetic metabolite and, in hypoxic conditions, hyperactivates lipid synthesis [156]. The nuclear localisation of acetyl-CoA synthetase 2 (ACCS2), which ligates acetate and CoA to produce acetyl-CoA, increases during limited amounts of oxygen, serum, or glucose. ACCS2, in complex with transcription factor EB, causes lysosomal biogenesis, autophagy, cell survival, and tumour growth in glioblastoma. The complex mediates the activation of lysosome- and autophagosome-related genes by producing acetyl-CoA for the promoter region of H3-histone acetylation, by using the acetate released from histone deacetylation [157]. ACSS2 has a direct role in de novo lipogenesis and the epigenetic regulation of gene expression involved in upregulating lysosomal biogenesis, autophagy, and lipid metabolism. An elevated expression of ACCS1/2 promotes histone H3 acetylation and FA synthase (FASN) expression in HCC patients [158]. FASN can undergo acetylation by KAT8 and deacetylation by HDAC3. FASN acetylation triggers its association with TRIM21, the ubiquitin ligase, which enhances its degradation, and is frequently reduced in HCC samples [159].

### 5.3. SREBPs and Lipogenesis

Sterol regulatory element-binding proteins (SREPBs) are a family of helix-loop-helix leucine zipper transcription factors that play a pivotal role in the transcriptional regulation of lipogenesis. SREBP activity is modulated post-translationally; for instance, the GSK3-mediated phosphorylation of SREBP targets it for degradation by the ubiquitin ligase SCFFBXW7 [160]. Protein arginine methyltransferase 5 (PRMT5) inhibits this phosphorylation through the modification of SREBP1a by R321 dimethylation. This modification impedes GSK3 interaction, leading to enhanced lipogenesis, which correlates with increased tumour proliferation and a poor prognosis in HCC patients [161]. Furthermore, FAO by peroxisome proliferator-activated receptors (PPARs) is fine-tuned through epigenetic modifications. PPARs and FAO are activated by the promyelocytic leukaemia protein in TNBC, which is accomplished by the reduction in PGC-1α acetylation [88].

### 5.4. Palmitoylation and Other Lipid Modifications

Palmitoylation, a type of fatty acylation, has an important role in different biological processes. Palmitic acid (PA)-induced palmitoylation, mediated by ZDHHC23, influences the degradation of PHF2, a tumour suppressor that targets the lipogenesis master regulator SREBP1c. The PHF2/SREBP1c axis emphasises the importance of monitoring dietary PA levels in HCC patients [162]. A palmitoyl transferase zinc finger DHHC-type palmitoyl transferase 3 (ZDHHC3)-mediated palmitoylation of PD-L1 blocks its ubiquitination and suppresses PD-L1-mediated immune evasion [163]. ZDHHC9 functions as a palmitoyl transferase for PD-L1 in breast cancer [164,165]. ZDHHC5 is transcriptionally upregulated in p53-mutant glioma, which mediates the palmitoylation of EZH2, affecting its methyl transferase activity, leading to lower levels of histone 3 lysine 27 trimethylation (H3K27me3), which contributes to malignancy and tumour progression [166]. There is a significant elevation of palmitoyl transferases in glioblastoma, which induces the palmitoylation of proteins that regulate cell survival and cell cycle in glioblastoma [167].

Lipid post-translational modification, like protein farnesylation, is essential for the activity of proteins such as GTPase Ras [168]. Farnesylation and geranylgeranylation, jointly referred to as prenylation, are a lipid post-translational modification which induces membrane association. The hippo pathway facilitates GGylation-dependent cell proliferation and migration in breast cancer [169,170]. GGylation promotes proliferation, migration, and invasion in gastric cancer, via the YAP signalling pathway [171].

### 5.5. Non-Coding RNAs in Lipid Metabolism

Non-coding RNAs (ncRNAs), including small RNAs, long non-coding RNAs (lncRNAs), micro RNAs (miRNAs), and circular RNAs (circRNAs), have emerged as key players in gene regulation. miRNAs regulate various aspects of lipid metabolism in cancer cells, including lipid synthesis, uptake, and oxidation. Seven key lipid metabolism-related miRNAs (LMRMs), including miR-148-3p and miR-375-3p, exhibit significant diagnostic potential for prognosis and diagnosis in prostate cancer (PCa). A TF-miRNA–mRNA regulatory network involving SNAI2, GATA3, and target genes like CLIC6 and SCNN1A, is significant in PCa progression and patient prognosis [172]. In HCC, miR-122 promotes lipid accumulation by targeting several genes involved in lipid oxidation, including CPT1A and acyl-CoA oxidase 1 (ACOX1) [173].

LncRNAs, on the other hand, interact with proteins involved in lipid metabolism or modulate the expression of lipid metabolism genes. The lncRNA HULC, prevalent in HCC, propels malignant development by activating ACSL1 and disrupting lipid metabolism. Its mechanism of action involves PPARa upregulation, miR-9 suppression via CpG methylation, and a cholesterol-driven positive feedback loop with RXRA [174]. A significant upregulation of lncRNA HOTAIR in NPC cells and clinical specimens was observed, with a positive correlation to FASN expression. HOTAIR knockdown resulted in FASN downregulation, suppressing NPC cell proliferation and invasion and inhibiting de novo synthesis of cellular free fatty acids. The downregulation of MMP-9 and p21 was also noted upon HOTAIR knockdown, suggesting HOTAIR’s crucial role in NPC progression and recurrence through FASN upregulation, making it a potential therapeutic target for NPC [175]. In NSCLC, the overexpression of lncRNA CASC19 significantly promotes cell proliferation and metastasis. CASC19 exerts its function by negatively regulating miR-301b-3p, leading to an increased expression of LDLR. The interaction between CASC19, miR-301b-3p, and LDLR establishes a regulatory axis influencing NSCLC progression [176]. In gastric cancer, the novel lncRNA FLJ22763 is downregulated and correlates with a lower histological grade and deeper invasion. FLJ22763 acts as a suppressor gene, inhibiting malignant behaviour in GC cells and tumour growth. Its reduced expression serves as a significant independent prognostic factor for GC, and it negatively regulates ACLY, influencing mRNA and protein levels [177].

**Table 2:** Lipid metabolism and epigenetic changes in cancer. An overview of the interplay between lipid metabolism and epigenetic changes in various types of cancer.

Lipid Metabolism Process	Epigenetic Changes	Cancer Type	Mechanism	References
PI3K-AKT- mTORC1 pathway	Histone Acetylation	Glioma, Prostate, Pancreatic Ductal Adenocarcinoma (PDAC)	AKT activation increases acetyl-CoA production, promoting histone acetylation. Upregulation in K-Ras-driven cancers.	[145,148,149]
ACLY Signaling	Histone Acetylation	Melanoma	ACLY enhances histone acetylation, promoting mitochondrial biogenesis.	[150]
β-Hydroxybutyrate (β-HB)	Histone Modification	Colorectal Carcinoma	Upregulated β-HB leads to histone β-hydroxy butyrylation, impacting p53 activity.	[152,153,154]
ACC1 Regulation	Histone Acetylation	Breast Cancer	Leptin and TGFβ signalling inhibit ACC1, affecting global histone acetylation.	[52]
Free Acetate Metabolism	Histone Acetylation	Glioblastoma	ACSS2 promotes lysosomal biogenesis and autophagy, impacting H3- histone acetylation.	[157]
FASN Acetylation	Histone Acetylation	Hepatocellular Carcinoma (HCC)	FASN acetylation regulates its degradation, reduced in HCC samples.	[159]
SREBPs Regulation via ubiquitination and methylation	Post-Translational Modifications of SREBPs	Hepatocellular Carcinoma (HCC)	PRMT5 prevents SREBP1a phosphorylation, influencing lipogenesis in HCC.	[161]
PPAR and FAO Activation	Acetylation Regulation	Triple-Negative Breast Cancer (TNBC)	PML protein activates PPAR and FAO by reducing PGC acetylation.	[178]
Palmitoylation	Histone Modification	Glioma	ZDHHC5 mediates palmitoylation of EZH2, affecting H3K27me3 levels.	[166]
Prenylation	Lipid modifications influencing protein function and potential epigenetic regulation	Breast Cancer, Gastric Cancer	Prenylation of proteins like Ras influences their membrane association and function in signalling pathways.	[169,170,171]
Lipid Metabolism	miRNA-Mediated Gene Regulation	Prostate Cancer (PCa)	LMRMs, including miR-148-3p and miR-375-3p, exhibit diagnostic potential for PCa.	[172]
Lipid Metabolism	lncRNA-Mediated Gene Regulation	Hepatocellular Carcinoma (HCC), Nasopharyngeal Carcinoma (NPC), Gastric Cancer	HULC activates ACSL1, affecting lipid metabolism. HOTAIR correlates with FASN expression. FLJ22763 acts as a suppressor in GC.	[174,175,177]

## 6. Conclusions and Future Prospects

Targeting the dysregulated lipid metabolism in cancer through the inhibition of transporters CD36 and FABP4, the disruption of de novo lipogenesis, and FAO enzymes, as well as cholesterol synthesis, has revealed potential avenues for therapeutics. Promising results from preclinical studies and ongoing clinical trials underscore the viability of these strategies. In addition, lipid rafts–specialised lipid domains within cell membranes—play a pivotal role in signalling pathways, important for tumour progression and metastasis. The dysregulation of cholesterol biosynthesis enzymes such as squalene synthase (SQS) contributes to lipid raft-associated signalling. Thus, disrupting lipid raft formation or inhibiting SQS impedes metastasis.

Cancer stem cells exhibit distinct metabolic characteristics, including enhanced de novo lipogenesis, lipid droplet accumulation, and FAO, which contribute to their stemness and aggressiveness compared to non-stem cancer cells. Key regulators of lipid metabolism in CSCs, such as SCD1 and SREBP1, offer promising therapeutic targets. Inhibitors of SCD1 and SREBP1, such as CAY10566 and A939572, hold potential to suppress lipid metabolism pathways critical for CSC survival and maintenance. Additionally, targeting FAO pathways or enzymes in the mevalonate pathway, such as HMG-CoA reductase, disrupts CSC energy balance and signalling pathways. Combining lipid metabolism inhibitors with conventional treatments like chemotherapy or radiotherapy may synergistically enhance antitumour effects and overcome therapy resistance.

Distinct patterns of lipid metabolism have emerged as potential diagnostic markers, offering a molecular fingerprint for specific cancer subtypes. These alterations correlate with disease progression, patient outcomes, and treatment response, thereby informing patient management strategies. Lipidomic profiling to develop reliable biomarkers for cancer classification is a promising approach that needs further investigation.

While significant progress has been made in understanding the role of an altered lipid metabolism in cancer, there are important considerations. For instance, while targeting lipid metabolism has shown promise with the inhibition of FA or cholesterol biosynthesis, compensatory mechanisms from dietary lipids, as well as the regulation of dynamic FAO, pose a significant challenge. As is the case with chemotherapeutic drugs, cancer anti-metabolic drugs can disrupt metabolic processes in normal cells. Thus, toxicity towards normal cells should be taken into account. Finally, it is important to take into account individual patient characteristics, tumour heterogeneity, and lipid patterns. Future studies focused on identifying specific targets and biomarkers based on lipidomic and metabolomic profiling will lead to the development of personalised treatment strategies in cancer. In this evolving landscape, the symbiotic relationship between epigenetics and metabolism in cancer has not only progressed significantly but also offers promising therapeutic horizons. The dynamic reversibility of epigenetic modifications, exemplified by breakthroughs like the EZH2 inhibitor tazemetostat, signifies a pivotal stride in epigenetics-based medicine. The crosstalk between epigenetic marks and metabolic reprogramming provides opportunities to fine-tune targeted strategies for cancer therapeutics.

## Figures and Tables

**Table 1:** Therapeutic targets in lipid metabolism. List of fatty acid metabolism targets and antibody or small-molecule inhibitors targeting them.

Target	Small-Molecule Inhibitor 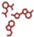	Antibody 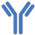
CD36	Sulfosuccinimidyl oleate (SSO) 2-methylthio-1,4-naphthoquinone (MTN)	JC63.1 Clone 1G04 Clone Ona-0-v1
FATP1	Lipofermata Arylpiperazine 5k (DS22420314)	-
FABP4	BMS309403 BD62694	-
ACC	Soraphen A ND-646 ND-654 5-tetracepoxy-2-furan acid (TOFA)	-
FASN	Cerulenin C75 Orlistat TVB-2640 GSK2194069	-
SCD	A-939572 CAY10566 CVT-11127	-
CPT1A	Etomoxir Perhexiline ST1326	-
ACSS/ASCL	VY-3-135 1-(2,3-di(thiophen-2-yl)quinoxalin-6-yl)-3-(2-methoxyethyl)urea Rosiglitazone	-
HMGCR	Statins	-
ACAT1	Avasimibe Avasimin	-
LXR	GW3965 LXR623	-
Cholesterol analogues	Rp1 (Ginsenoside)	-
